# Antibody signature induced by SARS-CoV-2 spike protein immunogens in rabbits

**DOI:** 10.1126/scitranslmed.abc3539

**Published:** 2020-06-08

**Authors:** Supriya Ravichandran, Elizabeth M. Coyle, Laura Klenow, Juanjie Tang, Gabrielle Grubbs, Shufeng Liu, Tony Wang, Hana Golding, Surender Khurana

**Affiliations:** Division of Viral Products, Center for Biologics Evaluation and Research (CBER), FDA, Silver Spring, Maryland, USA, 20871

## Abstract

Multiple vaccine candidates against SARS-CoV-2 based on viral spike protein are under development. However, there is limited information on the quality of antibody responses generated with these vaccine modalities. To better understand antibody responses induced by spike protein-based vaccines, we performed a qualitative study by immunizing rabbits with various SARS-CoV-2 spike protein antigens: S-ectodomain (S1+S2) (aa 16-1213), which lacks the cytoplasmic and transmembrane domains (CT-TM), the S1 domain (aa 16-685), the receptor-binding domain (RBD) (aa 319-541), and the S2 domain (aa 686-1213, lacking the RBD, as control). Resulting antibody quality and function were analyzed by enzyme linked immunosorbent assay (ELISA), receptor binding domain (RBD) competition assay, surface plasmon resonance (SPR) against different spike proteins in native conformation, and neutralization assays. All three antigens (S1+S2 ectodomain, S1 domain, and RBD), but not S2, generated strong neutralizing antibodies against SARS-CoV-2. Vaccination-induced antibody repertoire was analyzed by SARS-CoV-2 spike genome fragment phage display libraries (SARS-CoV-2 GFPDL), which identified immunodominant epitopes in the S1, S1-RBD, and S2 domains. Furthermore, these analyses demonstrated that the RBD immunogen elicited a higher antibody titer with 5-fold higher affinity antibodies to native spike antigens compared with other spike antigens; and antibody affinity correlated strongly with neutralization titers. These findings may help guide rational vaccine design and facilitate development and evaluation of effective therapeutics and vaccines against COVID-19 disease.

## Teaser

Understanding antibody epitope diversity and affinity generated by SARS-CoV-2 spike antigens in rabbits can aid COVID-19 vaccine development.

## INTRODUCTION

The ongoing pandemic of SARS-CoV-2 has resulted in more than 4.3 million human cases and 290,000 deaths as of 12^th^ May 2020 (*1*). Therefore, development of effective vaccines for prevention and medical countermeasures for treatment of SARS-CoV-2 infection is a pressing global priority. The spike glycoprotein has been identified as the key target for protective antibodies against both SARS-CoV-1 and SARS-CoV-2 (*2*–*5*). Consequently, multiple versions of the SARS-CoV-2 spike proteins are currently under evaluation as vaccine candidates using different modalities and delivery systems (*6*). However, only limited knowledge exists on antibody repertoire or quality of the immune response generated following vaccination by different spike vaccine antigens (*6*). Therefore, it is important to perform a comprehensive evaluation of post-vaccination antibody response to elucidate the quality of the immune responses elicited by spike-based vaccine candidates. This could also determine immune markers that may predict clinical benefit which would facilitate evaluation of vaccine candidates.

To better understand vaccination-induced antibody response in a pharmacological/toxicological animal model, we immunized rabbits with several SARS-CoV-2 spike proteins: spike-ectodomain (S1+S2), S1 domain, receptor-binding domain (RBD), and S2 domain, which lacks the RBD, as a control. Post-vaccination sera were analyzed by genome fragment phage display libraries covering the entire spike gene (SARS-CoV-2 GFPDL) to determine the polyclonal antibody epitope repertoire generated following vaccination as previously applied for other diseases (*7*–*11*). In addition, we used several antibody binding assays (ELISA, surface plasmon resonance (SPR) based real-time kinetics assay) (*11*–*13*) and an in vitro SARS-CoV-2 pseudovirion or wild-type virus neutralization assay to measure the quality and function of the antibodies elicited by the different SARS-CoV-2 spike antigens. This study could inform development and evaluation of SARS-CoV-2 vaccines and therapeutics based on the spike glycoprotein.

## RESULTS

### Rabbit immunization with SARS-CoV-2 spike antigens

**Fig. 1:**
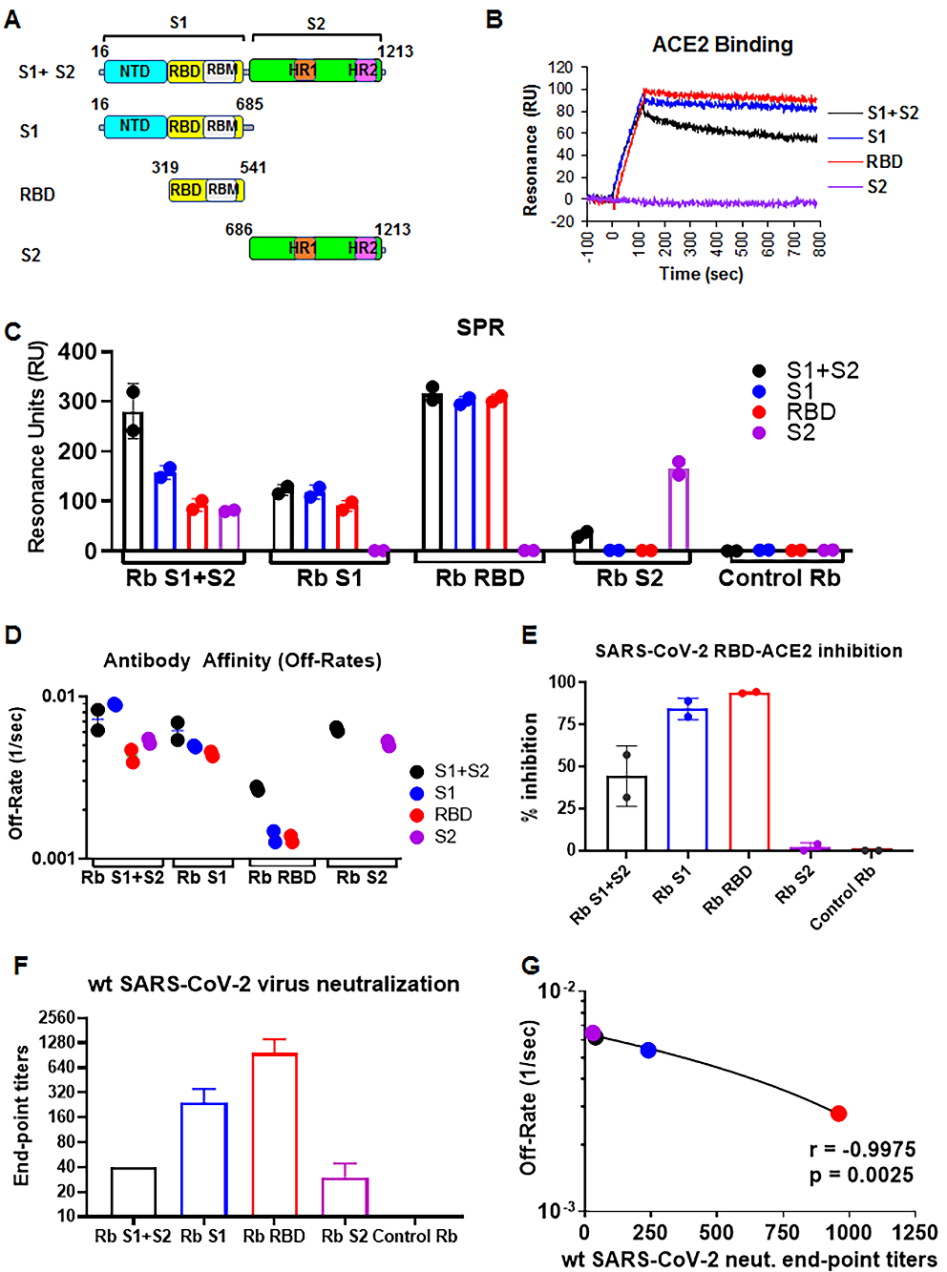
SARS-CoV-2 spike binding and SARS-CoV-2 neutralization by serum antibodies generated following rabbit immunization with spike antigens. A) Schematic representation of the SARS-CoV-2 spike protein and subdomains. Spike S1+S2 ectodomain (aa 16-1213) lacks the cytoplasmic and transmembrane domains (CT-TM), S1 domain (aa 16-685), RBD domain (aa 319-541), and S2 domain (aa 686-1213), all containing 6x His tag at C terminus, were commercially produced in either HEK 293 mammalian cells (S1 and RBD) or insect cells (S1+S2 ectodomain and S2 domain). The receptor-binding motif (RBM) encompasses residues 437-508. (B) Binding of purified proteins to human ACE2 (hACE2) proteins in SPR. Sensorgrams represent binding of purified spike proteins on low-density His-captured chips to 5 μg/mL human ACE2 protein. (C) SPR binding of antibodies from two rabbits each immunized twice with SARS-CoV-2 antigens to spike protein and domains from SARS-CoV-2 (S1+S2, black; S1, blue; RBD, red; and S2, purple). Total antibody binding is represented in maximum resonance units (RU) in this figure for 10-fold serum dilution. All SPR experiments were performed twice and the researchers performing the assay were blinded to sample identity. The variations for duplicate runs of SPR was <5%. The data shown are average values of two experimental runs. (D) Antibody off-rate constants were determined directly from the serum sample interaction with SARS-CoV-2 spike ectodomain (S1+S2), S1, S2, and RBD using SPR in the dissociation phase only for the sensorgrams with Max RU in the range of 20–100 RU. (E) RBD-hACE2 competition assay. Percent inhibition of hACE2 binding to RBD in presence of 1:50 dilutions of post-second vaccination rabbit serum was measured by SPR. (F) End-point virus neutralization titers for one rabbit from each group using wild type SARS-CoV-2 virus in a classical BSL3 neutralization assay based on CPE (Cytopathic effect) was performed as described in Materials and Methods. (G) Anti-spike ectodomain (S1+S2) binding antibody affinity as measured by antibody dissociation rates (off-rates) of post-vaccinated rabbit polyclonal antibodies correlated with the wt SARS-CoV-2 virus end-point neutralization titers (r = -0.9975, p <0.005). Pearson two-tailed correlations are reported for the calculation of correlations between anti-S1+S2 antibody affinity and end-point titers for one rabbit per immunogen. The color scheme in panel G is the same as in panel D/F.

Most spike-based vaccines currently under development are designed to contain the receptor-binding domain (RBD; aa 319-541) in some form. Therefore, we evaluated four different commercially available SARS-CoV-2 spike protein and subdomains: the spike (S1+S2) ectodomain (aa 16-1213), the S1 domain (aa 16-685), the RBD domain (aa 319-541), and the S2 domain (aa 686-1213), devoid of RBD, as a control (Fig. 1A, fig. S1). These spike proteins were either produced in HEK 293 mammalian cells (S1 and RBD) or insect cells (S1+S2 ectodomain and S2 domain). The purified S1+S2 ectodomain, the S1 domain, and the RBD proteins retained receptor-binding activity as demonstrated by SPR assay using human ACE2 (hACE2) protein, the SARS-CoV-2 receptor (Fig. 1B). The S1+S2 ectodomain, S1 domain, and RBD demonstrated high-affinity interaction with hACE2 with affinity constants ranging from 5.09 nM to 37.23 nM. The control S2 domain protein (purple curve), lacking the RBD, did not bind to hACE2, demonstrating specificity of this receptor-binding assay (Fig. 1B).

Female New Zealand white rabbits, a model frequently used to assess pharmacology/toxicology of vaccine antigens, were immunized twice intra-muscularly at a 14-day interval with 50 μg of the purified proteins (two animals each) mixed with Emulsigen adjuvant. Sera were collected before (pre-vaccination) and eight days after the first and second vaccination and analyzed for binding antibodies in ELISA and SPR, pseudovirion and wild-type SARS-CoV-2 virus neutralization assay, RBD competition assay, and by GFPDL analysis.

### Antibody response following immunization with different spike antigens

Serial dilutions of post-second vaccination rabbit sera were evaluated for binding of serum IgG to various spike proteins and domains in ELISA (S1+S2, black; S1, blue; RBD, red; and S2, purple) (fig. S2). Representative titration curves to spike ectodomain (S1+S2) and to the RBD in IgG-ELISA are shown in fig. S2A and B. End-point titers of the serum IgG were determined as the reciprocal of the highest dilution providing an optical density (OD) twice that of the negative control, no serum (fig. S2C). All four immunogens elicited strong IgG binding to the spike ectodomain (S1+S2). Binding to the individual domains (S1, S2, and RBD) was specific, in that sera generated by S2 vaccination bound to S2, but not to S1 or RBD, and vice-versa.

SPR allows antibody binding to captured antigens in real-time kinetics, including total antibody binding in resonance units (Max RU) and affinity kinetics. In ELISA, the antigens directly coated in the wells can be partially denatured, increasing the likelihood of presenting epitopes that are not exposed on the native forms of the proteins. In the SPR used here, the purified recombinant spike proteins were captured to a nickel- nitrilotriacetic acid (Ni-NTA) sensor chip to maintain their native conformation (as determined by hACE2 binding) to allow comparisons of binding to and dissociation from the four proteins (Fig. 1B). Importantly, the protein density captured on the chip surface is low (200 RU) and was optimized to measure primarily monovalent interactions, so as to measure the average affinity of antibody binding in the polyclonal serum (*9*, *14*). Additionally, whereas the ELISA measured only IgG binding, in SPR, all antibody isotypes contributed to antibody binding to the captured spike antigen.

As shown by SPR, all rabbit sera post-second vaccination contained anti-spike antibodies that were at least 80% IgG, with 10-15% contributions of IgA antibodies and minimal IgM (Fig. S3). Serial dilutions of post-vaccination serum were analyzed for binding kinetics with different spike proteins (shown in fig. S4A for S1+S2 binding at different serum dilutions). For antibody binding titers, maximum SPR signal measured for all post-second vaccination rabbit sera diluted 10-fold is shown in Fig. 1C. The spike ectodomain (S1+S2) generated antibodies that predominantly bound to S1+S2 (~280 RU), followed by the S1 protein (~157 RU), and 3-fold lower antibody binding to the RBD and the S2 domain (<100 RU) (Fig. 1C). The S1 domain antigen induced antibodies bound with similar titers (~100 RU values) to the S1+S2, S1 and RBD proteins, and did not show reactivity to the S2 domain. However, the antibody reactivity of rabbit anti-S1 serum to S1+S2 domain was at least 2-fold lower than the antibodies in the rabbit anti-S1+S2 serum. In contrast to S1, RBD immunization generated similar high-titer antibody binding to S1+S2, S1 and RBD (~300 RU), (Fig. 1C). The S2 domain induced antibodies that primarily bound to homologous S2 antigen (~153 RU) and only minimally bound to the S1+S2 ectodomain, and no binding to either S1 or RBD (Fig. 1C).

Antibody off-rate constants, which describe the fraction of antigen–antibody complexes that decay per second, were determined directly from the serum sample interaction with SARS-CoV-2 spike ectodomain (S1+S2), S1, S2, and RBD using SPR in the dissociation phase only for sensorgrams with Max RU in the range of 20–100 RU (fig. S4A) and calculated using the BioRad ProteOn manager software for the heterogeneous sample model as described previously (*12*). These off rates provide additional important information on the affinity of the antibodies following vaccination with the different spike proteins that are likely to have an impact on the antibody function in vivo, as was observed previously in studies with influenza virus, RSV and Ebola virus (*14*–*16*). Surprisingly, we observed substantial differences in the affinities of antibodies elicited by the four spike antigens (Fig. 1D and fig. S4B). Specifically, the RBD induced 5-fold higher affinity antibodies (slower dissociation rates) against S1+S2, S1 and RBD proteins, compared with the antibodies generated by the vaccination with the other three immunogens (Fig. 1D).

The functional activity of the antibodies elicited by the different spike proteins was evaluated in a RBD-hACE2 binding competition SPR assay (Fig. 1E), and in virus neutralization assays (Fig. 1F and fig. S5). In the RBD-hACE2 competition assay, the immune sera (at 1:50 serum dilution) from the RBD- and S1-vaccinated animals gave strong inhibition (mean 94% and 84%, respectively), whereas the S1+S2 post-vaccination sera demonstrated lower inhibition (mean 44%) of RBD-hACE2 interaction, indicating the immunofocusing of antibodies by RBD vaccination that block RBD-hACE2 receptor interaction.

SARS-CoV-2 neutralization was measured using SARS-CoV-2-FBLuc in a single-cycle pseudovirus neutralization assay in Vero E6 cells. The average percent inhibition by post-first and post-second rabbit vaccination are shown in fig. S5A. Pre-vaccination rabbit sera (Control Rb) did not neutralize SARS-CoV-2 in this assay. Sera generated by S1+S2-ectodomain, S1, and RBD (1:40 dilution) (but not anti-S2) showed 50-60% virus neutralization after a single vaccination, and 93-98% virus inhibition by the post-second vaccination sera (fig. S5A). Additionally, we determined the 50% end-point pseudovirus neutralization titers for the post-second vaccination rabbit sera. The RBD-immunized rabbits demonstrated 2-fold higher IC_50_ titers compared with spike ectodomain (S1+S2) and approximately 3-fold higher than S1 domain immune sera (Fig. S5B). Importantly, the end-point neutralization titers (PsVN50) strongly correlated with serum antibody affinity against the spike ectodomain (S1+S2) (r = -0.9012; p = 0.0022) (Fig. S5C).

Subsequently, we also conducted classical wild-type SARS-CoV-2 virus neutralization assay with 100 TCID_50_ of SARS-CoV-2 (strain USA-WA1/2020 isolate). Although the end-point titers of this assay were lower than the IC_50_ titers observed in the pseudovirus neutralization assay (Fig. 1F vs. fig. S5B), the RBD-immune serum clearly demonstrated higher neutralization end-point titer (>1:640) compared with the S1 or S1+S2 immune sera (1:160, and 1:40, respectively). As shown with the pseudovirus neutralization titers, a strong inverse correlation was observed between the virus neutralization end-point titers and the post-vaccination serum antibody affinity (r = -0.9975; p = 0.0025) (Fig. 1G). Therefore, in three different functional assays, the RBD-immune sera showed better functional activity which correlated with the higher affinity of the antibodies in the post-second vaccination rabbit sera.

### Epitope repertoires recognized by antibodies generated against SARS-CoV-2 spike antigens

**Fig. 2:**
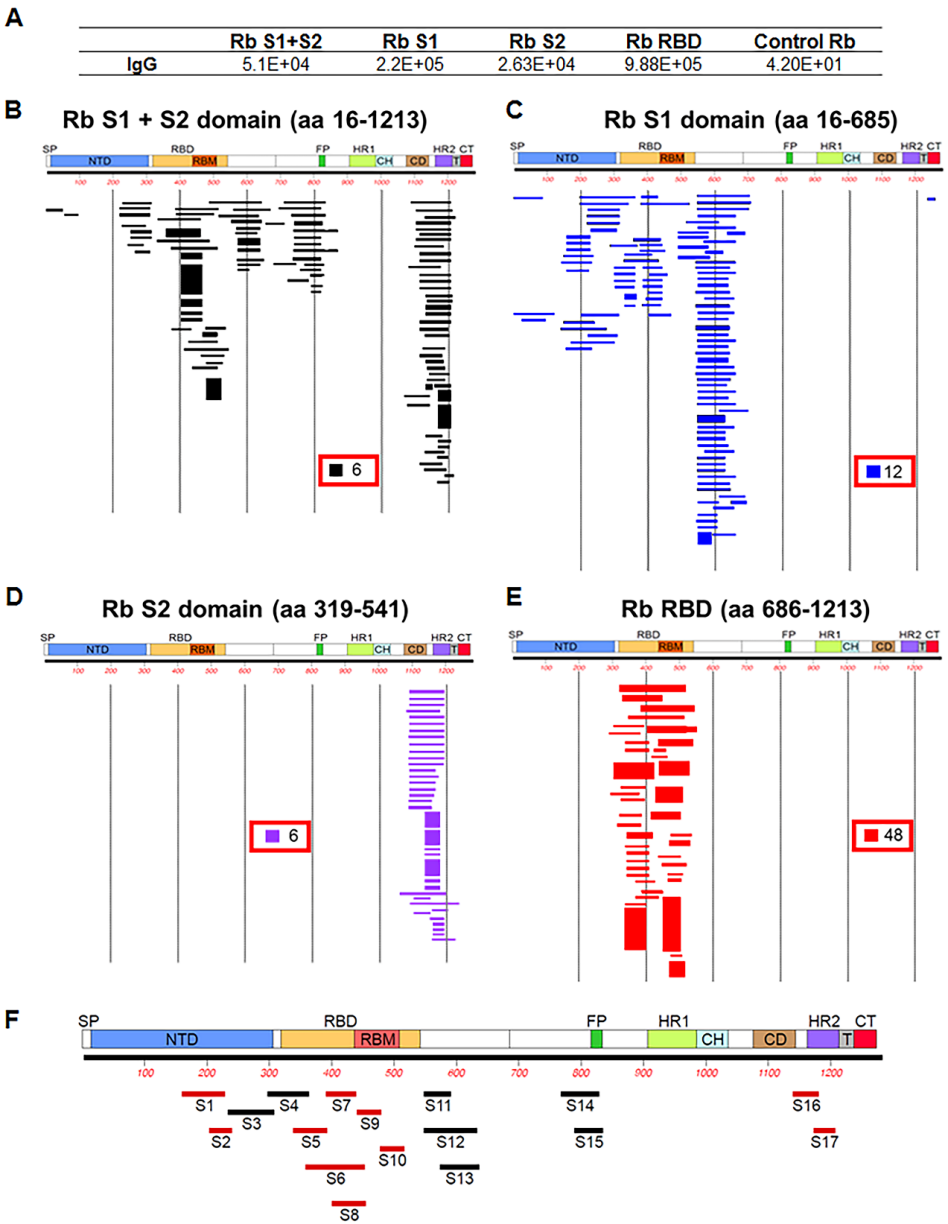
Antibody epitope repertoires generated by different SARS-CoV-2 spike antigens. (A) Number of IgG-bound SARS-CoV-2 GFPDL phage clones using the post-second vaccination rabbit polyclonal sera. (B-E) Graphical distribution of representative clones with a frequency of ≥2, obtained after affinity selection, and their alignment to the spike protein of SARS-CoV-2 are shown for the four vaccine groups: S1+S2 ectodomain (B), S1 (C), S2 domain (D) and S1-receptor binding domain (RBD) (E). The thickness of each bar represents the frequency of repetitively isolated phage, with the scale shown enclosed in a red box in the respective alignments in each panel. (F) Elucidation of the antibody epitope profile in SARS-CoV-2 spike following rabbit vaccination. Antigenic sites within the SARS-CoV-2 spike protein recognized by serum antibodies following rabbit vaccination (based on data presented in Fig. 1B-E). The amino acid designation is based on the SARS-CoV-2 spike protein sequence (fig. S4). The antigenic regions/sites are depicted below the spike schematic and are color coded. Epitopes of each protein are numbered in a sequential fashion indicated in black. The antigenic epitopes are color coded unique to this study (red bars) or if they were predicted by algorithms (black bars) previously by Grifoni *et al*. (*19*). Sequence residues for each antigenic site and their sequence conservation with other human coronaviruses is shown in table S1. The GFPDL affinity selection data was performed twice. Similar numbers of phage clones and epitope repertoire were observed in both phage display analyses.

The constructed SARS-CoV-2 GFPDL contains sequences ranging from 50-1500 bp long from the spike gene (GenBank #MN908947) with >10^7.2^ unique phage clones. The SARS-CoV-2-GFPDL displayed linear and conformational epitopes with random distribution of size and sequence of inserts that spanned the entire spike gene. SARS-CoV-2 GFPDL panning with individual post-second vaccination rabbit sera were conducted as described in Materials and Methods. The numbers of IgG-bound SARS-CoV-2 GFPDL phage clones with different serum samples ranged between 2.6 × 10^4^ to 9.8 × 10^5^/mL (Fig. 2A). Graphical distribution of representative clones with a frequency of ≥2, obtained after affinity selection, and their alignment to the spike protein of SARS-CoV-2 are shown for the four vaccine groups (Fig. 2 B-E). The spike (S1+S2) ectodomain induced diverse antibody response that included strong binding to epitopes in the C-terminal region of the soluble protein spanning the HR2 region (i.e., multiple phage clones with similar inserts). This region may not be highly exposed on native virions or infected cells but is clearly immunogenic in the soluble recombinant spike ectodomain. In addition, the rabbit anti-S1+S2 antibodies bound diverse epitopes spanning the RBD and to a lesser degree to the N-terminal domain (NTD), to the C-terminal region of S1, and to the N terminus of S2, including the fusion peptide (Fig. 2B and table S1). The S1 domain elicited very strong response against the C-terminal region of S1 protein and a diverse antibody repertoire recognizing the NTD and RBD regions (Fig. 2C and table S1). The recombinant RBD induced high-titer antibodies that bound the largest number of phages (9.88 x10^5^) (Fig. 2A) and were highly focused to the RBD/receptor binding motif (RBM) (Fig. 2E and table S1). Importantly, binding to long epitope sequences (most likely conformational epitopes) was observed predominantly with the anti-RBD sera but much less for the anti-S1+S2 or anti-S1 immune rabbit sera. In contrast, the recombinant S2 immunogen after two immunizations in rabbits elicited antibodies primarily targeting the C terminus of the S2 protein (CD-HR2).

All the immunodominant antigenic sites identified by the SARS-CoV-2 GFPDL panning of all four immune sera on the spike sequence are shown in Fig. 2F and in fig. S6. Alignment of the sequence with other coronaviruses shows that some of the antigenic sites are >70% conserved among several coronavirus strains isolated from humans and bats, especially sites located in the S2 domain (table S1). Structural depiction of these antigenic sites on the SARS-CoV-2 spike (fig. S7; in blue on PDB#6VSB) demonstrated that several of these antigenic sites identified here (primarily in and around RBD) are surface-exposed on the native prefusion spike (*3*).

## DISCUSSION

It is broadly accepted that an effective vaccine, which can be mass produced and deployed globally, will be required to curtail the current SARS-CoV-2 pandemic (*6*). Therefore, a better understanding of the humoral immune response generated by different vaccine antigens, including antibody epitope repertoire, antibody binding affinity, and functional activity, could greatly benefit the development and evaluation of vaccines against COVID-19.

In this study, we performed an in-depth evaluation of the antibody response generated by various SARS-CoV-2 spike antigens that are similar to the vaccine antigens being used in clinical development (*6*, *17*, *18*). A bioinformatics approach previously identified 279 potential B cell epitopes and 48 potential T cell epitopes in the spike glycoproteins of SARS-CoV, based on human antibody responses to SARS-CoV-1 infection and the corresponding epitopes in SARS-CoV-2 spike (*19*). Several of the predicted B cell epitopes overlapped with the sequences we identified in our GFPDL analysis: aa 287-317 in NTD-RBD overlaps with our antigenic site aa 298-363 which is 77% identical between SARS-CoV-1 and SARS-CoV-2; aa 524-598 and aa 601-640, in the C terminus of S1 overlap with our antigenic site containing aa 548-632 (78.8% conservation between SARS-CoV-1 and SARS-CoV-2); aa 802-819 in the S2 domain/FP overlaps with our antigenic site aa 768-828 (83% conserved between SARS-CoV-1 and SARS-CoV-2). Importantly, 10 out of 17 antigenic sites uniquely identified by the post-vaccination rabbit serum antibodies in the current study were missed by the prediction algorithms probably. This could be influenced by differences between antibody responses in rabbits and humans or may be due to sequence differences between SARS-CoV-1 and SARS-CoV-2, underscoring the limitations of the predictive algorithms in the in silico approach. However, confirmation of exposure of these antigenic sites on the trimeric form of the SARS-CoV-2 spike and more importantly, on SARS-CoV-2 virions, will requires further investigation. One of the possible limitations of GFPDL-based assessments is that although the phage display is likely to detect both conformational and linear epitopes on the CoV-2 spike proteins, they are unlikely to detect paratopic interactions that may require post-translational modifications, or quaternary epitopes formed by cross-protomers.

Surprisingly, the S2 domain does not appear to elicit as many neutralizing antibodies as RBD or S1. Although S2 contains the fusion peptide, it does not appear to be as immunogenic, compared with S1 or RBD, in generating binding antibodies to the intact spike (S1+S2) ectodomain, as observed in both IgG ELISA and SPR. It is possible that in addition to the RBD, additional antigenic sites, for example aa residues 548-632 located close to the RBD in the S1 domain C terminus, may also contribute to neutralizing potential of antibodies.

Even though we characterized the purified proteins in various assays, there is a possibility that the structure of the antigens used in the study is different from the corresponding authentic spike protein on the surface of SARS-CoV-2 virion particle. These commercial antigens were also manufactured in different systems (insect or mammalian cells), so differential glycosylation and other modifications may be present. This study used two rabbits per immunogen and was designed to be qualitative rather than quantitative. It will be important to investigate how the vaccination-induced immune responses in rabbits compare with immune responses in humans who are either vaccinated with candidate vaccines or COVID-19 survivors. Moreover, the rabbits were not challenged with virus, so we cannot determine whether the antibodies induced by vaccination were protective.

One unexpected finding in this study was the higher affinity of antibodies elicited by the RBD compared with the other spike antigens (S1+S2 ectodomain, S1 and S2 domains), which correlated strongly with the neutralization titers. In studies related to other viral infections, high affinity antibody responses were associated with clinical benefit in model systems or infected individuals (13-14, 19-20). Thus, vaccines that can elicit high affinity antibodies may provide a considerable advantage for clinical outcome of SARS-CoV-2 infection and contribute to amelioration of disease in infected individuals. Therefore, in addition to measurements of antibody binding titers and virus neutralization, this and the previous studies demonstrate the importance of assessments of antibody affinity maturation during SARS-CoV-2 vaccine trials. In addition to designing the best immunogen, selection of appropriate adjuvant could drive responding B cells into affinity maturation in germinal centers, as previously observed in clinical trials of influenza vaccines (*10*, *11*)*.*

In summary, our study highlights the need to perform comprehensive analysis of immune response generated following vaccination or SARS-CoV-2 infection to identify biomarkers of protective immunity. In depth understanding of quantitative and qualitative aspects of immune responses generated by different spike protein vaccine antigens could aid the development and evaluation of effective SARS-CoV-2 therapeutics and vaccines.

## MATERIALS AND METHODS

### Study design

The objective of this study was to investigate humoral immune response following vaccination with different SARS-CoV-2 spike immunogens. We used purified recombinant spike antigens to immunize female New Zealand White rabbits. Due to time, funding, and staffing restrictions during the COVID-19 pandemic, we began the exploratory study with 8 rabbits and maximized the number in each group (n=2). Using ANOVA, to compare between the two groups with sample size of 2, we needed a power of 0.0952 to achieve an effect size of 0.5 with significance level of 0.05. Samples and assays were run in duplicate or triplicate when possible after consideration of ethical animal sampling guidelines. Research fellows running the antibody assays were blinded to the identity of the groups for assessments of outcomes. Primary data are reported in data file S1.

### Recombinant CoV proteins

Recombinant SARS-CoV-2 proteins were purchased from Sino Biologicals (S1+S2 ectodomain; 40589-V08B1, S1; 40591-V08H, RBD; 40592-V08H or S2; 40590-V08B). Recombinant purified proteins used in the study were either produced in HEK 293 mammalian cells (S1 and RBD) or insect cells (S1+S2 ectodomain and S2 domain).

### Rabbit immunization studies

Female New Zealand white rabbits (Charles River labs) were immunized twice intra-muscularly at 14-day intervals with 50 μg of purified recombinant proteins mixed with Emulsigen adjuvant (MVP Adjuvants). Two animals were used per immunogen. All animal experiments were approved by the U.S. FDA Institutional Animal Care and Use Committee (IACUC) under Protocol #2008-10. The animal care and use protocol meets National Institutes of Health guidelines. Sera were collected before (pre-vaccination) and 8 days after the first and second vaccination and analyzed for binding antibodies in ELISA, SPR, neutralization assay and GFPDL analysis.

### ELISA

96 well Immulon plates were coated with 100 ng/100 μL of recombinant spike immunogens (protein and protein domains) in PBS overnight at 4°C. Starting at a 1:100 dilution, serum samples were serially diluted 1:5 and applied to the protein-coated plate in 10 μL for 1 hour at ambient temperature. Serum samples were assayed in duplicate. Naïve serum samples were assayed along with the experimental samples. After three washes with PBS/0.05% Tween 20, bound antibodies were detected with a donkey anti-rabbit IgG Fc-specific HRP-conjugated antibody (Jackson Immuno Research) After 1hr, plates were washed as before and o-Phenylenediamine dihydrochloride (OPD) was added for 10min. Absorbance was measured at 492 nm. End titer was determined as 2-fold above the average of the absorbance values of the no serum control. The end titer is reported as the last serum dilution that was above this cutoff.

### Antibody binding kinetics of post-vaccination sera to recombinant SARS-CoV-2 proteins by SPR

Steady-state equilibrium binding of post-vaccination rabbit polyclonal serum was monitored at 25°C using a ProteOn surface plasmon resonance (BioRad). The purified recombinant spike proteins were captured to a Ni-NTA sensor chip with 200 resonance units (RU) in the test flow channels. The native functional activity of the spike proteins was determined by binding to the 5 μg/mL human ACE2 protein (Fig. 1B).

For serum SPR analysis, the protein density on the chip was optimized to measure monovalent interactions independent of the antibody isotype. Serially diluted (10-, 20-, 40-, 80-, and 160-fold) freshly prepared sera were injected at a flow rate of 50 μl/min (120 s contact duration) for association, and disassociation was performed over a 600 s interval. Responses from the protein surface were corrected for the response from a mock surface and for responses from a buffer-only injection. SPR was performed with serially diluted serum of each animal in this study.

Antibody isotype analysis for the SARS-CoV-2 spike protein-bound antibodies in polyclonal serum was performed using SPR. Total antibody binding was calculated with BioRad ProteOn manager software (version 3.1). All SPR experiments were performed twice and the researchers performing the assay were blinded to sample identity. In these optimized SPR conditions, the variation for each sample in duplicate SPR runs was <5%. The maximum resonance units (Max RU) data shown in the figures was the RU signal for the 10-fold diluted serum sample. Antibody off-rate constants, which describe the fraction of antigen–antibody complexes that decay per second, are determined directly from the serum/ sample interaction with SARS CoV-2 spike ectodomain (S1+S2), S1, S2, and RBD using SPR in the dissociation phase only for the sensorgrams with Max RU in the range of 20–100 RU and calculated using the BioRad ProteOn manager software for the heterogeneous sample model as described before (*12*). Off-rate constants were determined from two independent SPR runs.

### SARS-CoV-2 pseudovirus production and neutralization assay

Human codon-optimized cDNA encoding SARS-CoV-2 S glycoprotein (NC_045512) was synthesized by GenScript and cloned into eukaryotic cell expression vector pcDNA 3.1 between the *BamH*I and *Xho*I sites. Pseudovirions were produced by co-transfection Lenti‐X 293T cells with pMLV-gag-pol, pFBluc, and pcDNA 3.1 SARS-CoV-2 S using Lipofectamine 3000. The supernatants were harvested at 48h and 72h post transfection and filtered through 0.45-mm membranes.

For the neutralization assay, 50 μL of SARS-CoV-2 S pseudovirions were pre-incubated with an equal volume of medium containing serum at varying dilutions at room temperature for 1 hour, then virus-antibody mixtures were added to Vero E6 cells in a 96-well plate. After a 3 hours incubation, the inoculum was replaced with fresh medium. Cells were lysed 48 hours later, and luciferase activity was measured using luciferin-containing substrate. Controls included cell only control, virus without any antibody control and positive control sera.

### Classical wild-type SARS-CoV-2 virus neutralization assay

100 TCID50 of SARS-CoV-2 (isolate USA-WA1/2020) was incubated with 2-fold serial rabbit serum dilutions in a round bottom plate at 37°C for 1 hour. The virus-antibody mixture was then added to a 96-well plate with 5x10^4^ Vero E6 cells. After 1 hour the mixture was removed and replenished with fresh MEM containing 2% FBS. Cells were incubated at 37°C for an additional 72 hours, then cytopathic effect (CPE) was measured by CellTiter-Glo luciferase assay (Promega). Luciferase abundance was determined using Veritas luminometer. The end-point titers were calculated as the last serum dilution resulting in at least 50% SARS-CoV-2 neutralization.

### SPR-based RBD-hACE2 binding and inhibition assay

The recombinant SARS-CoV-2 RBD protein from HEK 293 cells (RBD) was captured on a sensor chip with 500 resonance units (RU) in the test flow channels. Samples of 200 μl of freshly prepared post-second immunization rabbit sera at a 10-fold dilution were injected at a flow rate of 50 μl/min (contact duration, 180 s) for association. Following antibody binding, recombinant human ACE2 (Acro biosystems; 1 μg/mL) was injected at a flow rate of 50 μl/min (contact duration, 120 s) for association. Responses from the protein surface were corrected for the response from a mock surface free of protein and for responses from a buffer-only injection. Pre-vaccination animal sera were used as a negative control. Total hACE2 binding and data analysis and % inhibition by immune sera were calculated with Bio-Rad ProteOn Manager software (version 3.0.1).

### GFPDL construction

The SARS-CoV-2 spike gene (GenBank #MN908947) was chemically synthesized (GenScript) and used for cloning and construction of phage display libraries. A gIII display-based phage vector, fSK-9-3, was used where the desired polypeptide can be displayed on the surface of the phage as a gIII-fusion protein. Purified DNA containing the spike gene was digested with *DNase I* to obtain gene fragments of 100-1000 bp size range and used for GFPDL construction as described previously (*7*–*9*). The phage libraries constructed from the SARS-CoV-2 spike gene display viral protein segments ranging in size from 30 to 350 amino acids as fusion proteins on the surface of bacteriophage.

### Affinity selection of SARS-CoV-2 GFPDL phages with polyclonal rabbit serum

Prior to panning of GFPDL with polyclonal serum antibodies, serum components that could non-specifically interact with phage proteins were removed by incubation with UV-killed M13K07 phage-coated petri dishes (*9*). Equal volumes of each post-vaccination rabbit serum were used for GFPDL panning. GFPDL affinity selection was carried out in-solution with protein A/G (IgG) specific affinity resin as previously described (*7*, *8*, *10*). Briefly, individual rabbit serum was incubated with the GFPDL and the protein A/G resin and the unbound phages were removed by PBST (PBS containing 0.1% Tween-20) wash followed by washes with PBS. Bound phages were eluted by addition of 0.1 N Gly-HCl pH 2.2 and neutralized by adding 8 μL of 2 M Tris solution per 100 μL eluate. After panning, antibody-bound phage clones were amplified, the inserts were sequenced, and the sequences were aligned to the SARS-CoV-2 spike gene to define the fine epitope specificity in the post-vaccination rabbit sera. The GFPDL affinity selection was performed in a blinded fashion. Similar numbers of bound phage clones and epitope repertoire were observed in the two GFPDL panning experiments.

### Sequence and structural alignments

Spike protein sequences of SARS-CoV-2 (GenBank#MN908947), SARS-CoV-1 BJ01 strain (GenBank#AAP30030.1), MERS CoV KOR/KNIH/2015 (GenBank#AKN11075.1), Bat SARS-like CoV ZC45 (GenBank#AVP78031.1), Bat SARS-like CoV ZXC21 (GenBank#AVP78042.1), Bat CoV BM48-31/BGR/2008 (GenBank#ADK66841.1), Human CoV 2c EMC/2012 (GenBank# AFS88936.1), Human CoV NL63 (NCBI#YP_003767.1), and Human CoV HKU1 (NCBI#YP_173238.1) were used for alignment. Structural alignments were depicted on SARS-CoV-2 Spike prefusion structure PDB#6VSB (3).

### Statistical Analysis

Correlations were calculated with the Pearson method and P value for correlation was calculated by two-tailed test.

## SUPPLEMENTARY MATERIALS

stm.sciencemag.org/cgi/content/full/scitranslmed.abc3539/DC1 **Figure S1:** Purified SARS-CoV-2 proteins analyzed by SDS-PAGE under reducing and non-reducing conditions. **Figure S2:** Anti-spike reactivity of post-vaccination rabbit sera measured by ELISA. **Figure S3:** Isotyping of rabbit serum binding to the SARS-CoV-2 spike protein following immunization. **Figure S4:** Steady-state equilibrium analysis of serum antibody binding by SPR. **Figure S5:** Neutralizing activity of post-vaccination rabbit serum antibodies in a pseudovirion neutralization assay. **Figure S6.** Sequence alignment of spike protein from diverse CoV strains. **Figure S7.** Structural representation of antigenic sites identified in SARS-CoV-2 using GFDPL. **Table S1.** Sequence conservation of antigenic sites among different CoV strains **Data file S1.** Primary data
